# Evaluation of Pore-Former Size and Volume Fraction on Tape Cast Porous 430 Stainless Steel Substrates for Plasma Spraying

**DOI:** 10.3390/ma17225408

**Published:** 2024-11-05

**Authors:** Yifei Yan, Olivera Kesler

**Affiliations:** Department of Mechanical and Industrial Engineering, University of Toronto, 5 King’s College Road, Toronto, ON M5S 3G8, Canada; yf.yan@mail.utoronto.ca

**Keywords:** sintering, 2D qualitative analysis, stainless steels, bulk diffusion, metal-supported solid oxide cell

## Abstract

Porous 430L stainless steel disks made by tape casting with various pore-former sizes and volume fractions were evaluated as substrates for solid oxide cell (SOC) fabrication by plasma spraying. This work reports the substrate properties relevant to the SOC operation of disks made by using extra fine metal powder with dense sintering to minimize the fine porosity between particles. In contrast, the coarse porosity is introduced by the pore former. We found that the 60 μm pore former at a 45 vol% fraction has the best application fit; it gives an adequate gas permeability of 3.11 × 10^−13^ m^2^ and an average open pore size of 45.90 μm. Compared to a commercial substrate with a similar porosity perimeter/steel area ratio, the porosity and gas permeability are 1.6 and 3 times higher, respectively. The detected maximum surface pore is 49 μm, allowing gas-tight electrolytes fabricated by plasma spray deposition.

## 1. Introduction

Fuel cell technologies have proven effective in converting energy between chemical and electrical forms without emissions. Among those technologies, solid oxide cells (SOCs) operate at high temperatures, typically from 650 °C to 800 °C [1,2], and have the advantage of being able to use both hydrogen and carbon-containing fuels [3] plus the highest efficiencies of any full cell type, approximately 60% [1,3]. Conventional SOCs are ceramic-based, with one of the functional layers made thick enough to support the other cell layers mechanically [4]. Metal-supported solid oxide cells (MS-SOCs), on the other hand, utilize a metal layer to support the electrochemically active cell layers, offering the advantage of lower material cost and reduced ramping time from hours to tens of minutes [5,6] due to high material fracture toughness and high thermal conductivity [7]. Some metal support requirements include the following: (1) the thermal expansion coefficient of the ceramic layers and support layer needs to be matched, especially for the electrolyte layer (typically 8 mol% yttria-stabilized zirconia, TEC_YSZ_~10.5 ppm K^−1^) [8]; (2) it must allow gas diffusion to the electrodes; (3) it must be oxidation resistant; (4) the support layer should not react or have interdiffusion with other cell layers [9]. One of the options for the support layer is 430L stainless steel because the TEC (11.4 ppm K^−1^ [10]) is close to YSZ’s and has adequate corrosion resistance.

When fabricating SOCs, the most used technologies are wet-ceramic-based, such as tape casting [11], gel casting [12], and screen printing [13]. These methods require at least three wet ceramic steps and often two sintering steps, each taking approximately one day to complete [14]. Plasma spraying, on the other hand, is a process where it takes minutes to complete each cell layer deposition, with no subsequent sintering step required [15]. It is a proven technology, making thermal barrier coatings on turbine blades [16], and has also been studied for use in SOC layer fabrication [17,18]. To apply the plasma spraying method, the substrate surface should have adequate roughness and no large pores (ideally <40 µm, no more than 60 µm) that can cause defects in the subsequently deposited electrolyte layer [19,20]. Combining metal support with plasma spraying together can reduce SOC cost by approximately 50% due to the rapid processing and the use of a less expensive material for the support structure [21,22]. However, metal supports introduce additional challenges, including oxidation [23] and Cr poisoning or interdiffusion with the adjacent electrode layer [24]. The porous substrate can be engineered with a low surface-area-to-volume ratio to increase cell durability to slow down oxidation. It can be further protected from oxidation or interdiffusion with the electrode by applying additional coatings on material surfaces [25,26,27].

**Figure 1:**
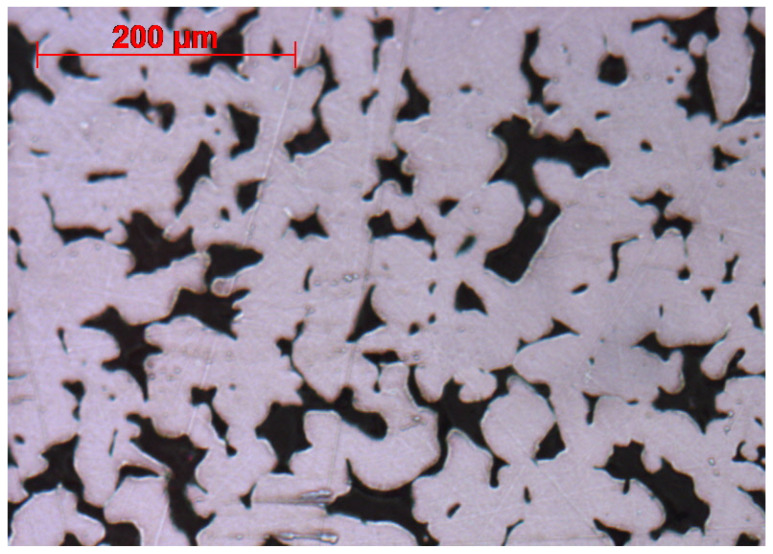
Conventional metal substrates (MG1, Mott Corp) with irregularly shaped porosity showing small steel features and small porosity such as those shown in the red circles.

Most metal-supported SOCs use supports sintered from metal powder with a size of 10 s of µm [28,29,30,31]; due to the large particles and difficulty to achieve final sintering stage, pore formers may not be required to obtain porosity for gas transport. A metal support created from this large particle shows some small porosity and small steel features (fingers) as shown in Figure 1. These small steel fingers are the weakest points where breakaway oxidation can happen. This is due to local chromium depletion [32,33] where iron oxide grows and blocks nearby porosity. Even without breakaway oxidation, the narrow porosity can also be blocked by chromium oxide scale formation [34]; it also adds up the surface area to the volume of the steel scaffold. This type of support is common in both in-house-made and commercial substrates [35,36,37,38,39,40].

In a previous study, a new strategy of using fine steel particles for dense sintering and large spherical pore-former particles effectively reduced the surface area susceptible to oxidation [41]. This work continues to investigate the impact of various pore-former volume fractions and sizes on the sintered porous material. The evaluation metrics include gas permeability before and after oxidation in air, normalized mass gain after oxidation, open porosity size distribution and volume fraction, surface roughness, and perimeter-to-area ratio.

Jie Lin et al. reported a new 430L metal support, fabricated by the phase inversion method to create aligned porosity using a powder size and sintering condition (d_0.5_ = 10 µm, 1250 °C, 3.5 h) [42]. Despite the high-power density of 1079 mW/cm^−2^ and 6 × 10^−11^ m^2^ permeability obtained, however, the resulting structure of the non-channeled part is still loose, with lots of powder packing porosity left, and there is no long-term operation result reported. Jason Lee et al. [43,44] demonstrated tape casting and sintering to make a porous mass transport layer used in PEMEC which also emphasizes mass transport, but the results still show much dead-end fine porosity with 37.6% porosity and a 0.15 perimeter-to-area ratio by image analyzing their samples (5 µm titanium powder). Their 60 µm pore-former sample has 43.8% porosity and a 0.11 perimeter-to-area ratio. So, besides SOC application, this sintering strategy can also benefit PEMEC as a porous mass transport layer but will also benefit the denser structure proposed by this work. Steven Pirou et al. developed a cell stack with a very high volumetric power density of up to 5.6 kW/L [45], but the utilized metal support only has about 10% porosity. The monoliths were sintered at 1290 °C for 6 h to sinter both the ceramic electrolyte and porous steel support. The porosity can potentially be improved by using a larger pore former and increasing the coordination number around pores as discussed below.

## 2. Materials and Methods

**Figure 2:**
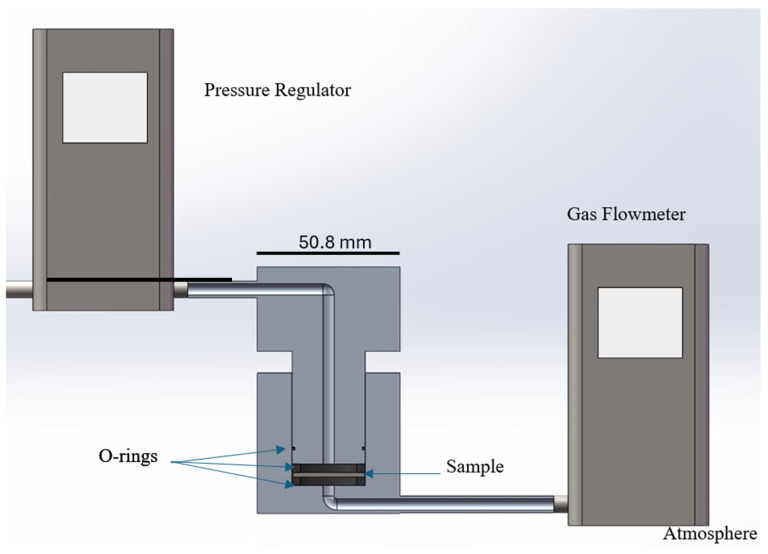
Schematic of in-house-made gas permeability measuring fixture. The fixture is made of two machined aluminum pieces with gas flow channels to clamp the substrate between two rubber O-rings, which prevents side gas leaks. Compressed air flow through the sample is measured as a function of pressure difference controlled by a pressure regulator.

The metal powder used for this study is medium chromium alloy 430L (Sandvik Osprey Ltd., Neath, UK); the pore formers are Poly(methyl-methacrylate) (PMMA) beads with four sizes: d_50_ = 20 μm, 40 μm (Lamberti, Skedsmokorset, Norway), 60 μm, and 90 μm (Huaqing Natural Ingredients, Xi’an, China). The elemental composition and size distribution of 430L SS powder are as follows: 16 wt.% Cr, 0.7 wt.% Mo, 0.60 wt.% Si, balance Fe; d_0.1_ = 2.93 µm, d_0.5_ = 5.67 µm, and d_0.9_ = 10.72 µm. The particle size distribution of the powders was analyzed by a laser particle size analyzer (Mastersizer 2000, Malvern Panalytical, Malvern, UK). The results are shown in Figure 2. The tape casting slurry has the following composition: 5 mL 18.9 wt% PVA (98–99% hydrolyzed low molecular weight, Thermo Scientific, Waltham, MA, USA) solution, 7.5 mL powders, 0.5 mL 50 wt% PEI solution (M_w_~2000, Sigma Aldrich, St. Louis, MO, USA), 0.25 mL antifoam 204 (Sigma Aldrich), and 5 mL distilled water, followed by 18 h ball milling and vacuum de-bubbling before tape casting at 1 mm blade height with a moving speed of 2 mm/s. Additional details on slurry composition development can be found in previous work [41].

To measure the substrate shrinkage relative to green tape, the size of rectangular tape strips was recorded as *L*_o_, with width parallel to the casting direction and length perpendicular to the casting direction. The sintered substrate sizes were recorded as *L*. The shrinkage *S* was then calculated using Equation (1):

*S* = 1 − *L*/*Lo* (1)

A stylus profilometer (D120, AlphaStep, Milpitasn, CA, USA) measured surface profiles with 7 parallel 2.5-mm-long scans separated 0.5 mm at 0.1 mm/s and 5 mg contact force. The surface roughness R_a_ evaluates how the surface height profile deviates from the mean value:

(2) $$R_{a}=1/l\;\int_{0}^{l}|y(x)|dx$$

In Equation (2), *l* is the length of the evaluated profile and |y(x)| is the height difference between the measured height and mean value at position x. A python script was used to find the average pit width and depth for those pits deeper than 10–30 µm depending on the pore-former size (10 µm for PF20, 20 µm for PF40, 30 µm for PF60 and PF90). Those numbers would give more insight into the potential electrolyte deposition defects locally, while R_a_ better describes the average profile.

Image analysis measured the substrate porosity and porosity-perimeter-to-steel-area ratio using cross-sessional images and ImageJ software (1.54f 29). The images were obtained using an optical microscope (Axio Scope, Zeiss, Oberkochen, Germany) and then converted to binary with a threshold greyscale cut-off. Porosities were approximated by counting total dark pixels that represent porosity over the number of total pixels, and porosity-perimeter-to-area ratios were calculated by using mean porosity perimeter multiplied by the number of pores, then divided by the bright area that represents steel.

Gas permeability was measured by an in-house fixture with a schematic shown in Figure 2. The substrate was clamped between two square silicone rubber O-rings. The permeation area diameter was 19 mm. A pressure controller (PCD-5PSIG-D5P, Alicat Scientific, Tucson, AZ, USA) was used to apply various pressures from one side, and a gas flow meter was connected to the atmosphere (M-50SCCM-D/5M, Alicat Scientific, Tucson, AZ, USA) to measure the gas flow rate through substrates. Five points from 0 kPa to 4.1 kPa were measured, and at least three substrates with the same pore former composition were tested. Darcy’s law (3) was used to calculate the gas permeance of the empty sample holder and holder with the substrate. Substrate permeability *k* was later calculated:

*P* = *q*/(Δ*p*×*μ*) (3)

*P_s_* = *P_t_* × *P_e_*/(*P_e_* − *P_t_*) (4)

*k* = *P_s_* × *A*/*L* (5)

*P* is permeance; *s*, *e*, *t* subscripts note the permeance of substrate, empty fixture, and fixture with a substrate, respectively; *q* is volumetric gas flow rate; Δ*p* is pressure difference over substrates; *μ* is the dynamic viscosity of air; *k* is substrate permeability; *A* is permeation area; *L* is substrate thickness. Both permeability before and after a 900 °C-24 h oxidation in the atmosphere were measured.

In addition to gas permeability, substrate mass gain was recorded and normalized by area:

(6) $$\dot{m}=\Delta m/(r\times V(1-p))$$

*p* is porosity; *V* is substrate bulk volume; *r* is porosity-perimeter-to-steel-area ratio; Δ*m* is a mass gain of the substrate after oxidation; $\dot{m}$ is normalized mass gain by area.

Additional MG1 commercial stainless-steel filters are included as conventional support as a comparison. These supports show a structure similar to the common existing solution, and we can experiment with more measurements rather than doing image analysis from other work.

## 3. Results

### 3.1. Particles and Substrate Geometry

**Figure 3:**
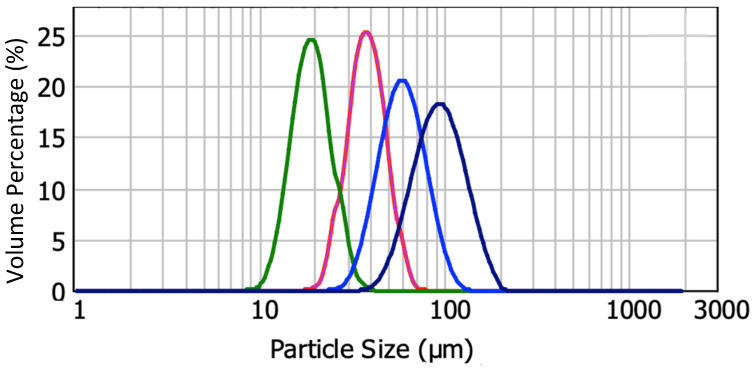
Particle size distribution data for four sizes of PMMA pore former. From left to right, they are PF20, PF40, PF60, and PF90.

Figure 3 shows all pore-former particle size distributions measured by the laser diffraction method. It measures each particle’s size based on the diffraction caused by the suspended particles and displays the results by showing the volume percentage distribution of corresponding particle sizes. The particle size measurement ranges from 0.02 µm to 2000 µm. In Figure 3, all of the curves have single symmetrical peaks. This distribution means small particles are more frequent, but there is a tail with fewer, much larger particles. The steel particle distribution was reported in previous work [41] and more details can be found in Figure S1. A detailed particle size distribution of pore formers can be found in Figures S2–S5.

In Table 1, PF20 and FP40 have a similar span of about 0.6, while PF60 and PF90 are closer to 0.8. Both suppliers provide powder with a narrow powder size distribution. The steel powder size distribution is not symmetrical. The difference between d_0.9_ and d_0.5_ is greater than the difference between d_0.1_ and d_0.5_, and this shows that the coarser powder has a larger size distribution than the fine powder, potentially caused by powder agglomeration.

**Figure 4:**
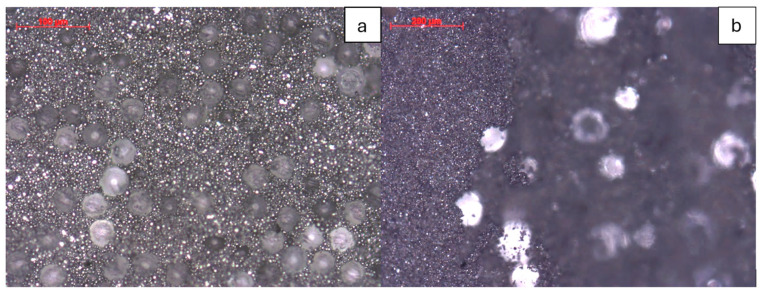
Optical microscopy image of the as-cast PF40 tape top side showing spherical metal powder and PMMA beads (**a**). An optical microscope image of as-cast PF90 tape on the plate side shows a flat layer of steel powder covering the PMMA beads on the left side, while the right part of the image has the steel powder layer removed, which is out of focus in the background (**b**).

Both suppliers’ steel particles and pore formers have spherical shapes, as shown in optical microscope images in Figure 4. The as-cast green tapes are flat, rigid, and crumble easily without much deformation by hand. For smaller pore former sizes like PF20, both the air (top) and plate (bottom) sides show uniform pore former distribution, but as the particle size increases, the plate side starts to show less pore former, and a thin layer of steel powder covers the plate side. This issue is most prominent for PF90 tapes, as shown in Figure 4b. The left side of Figure 4b shows a dense layer of steel powder that is in focus; on the right side, there are blurry pore formers out of focus that are located in the plane further away from the focal plane. The tape’s plate side with less pore former has less open porosity after sintering, reducing gas permeability.

The lack of a large pore former on the plate side is possibly due to the larger PMMA particles extending through multiple laminar flow regions during the tape casting process, resulting in the particles experiencing additional lateral shear force from the doctor blade motion, pulling the larger pore former particles along the casting direction. When the larger, lower-density PMMA particles encounter smaller, higher-density steel particles near the slurry bottom, they roll over the steel particles, resulting in a net upward motion that leaves the bottom portion of the tape devoid of the large pore-former particles. However, smaller pore former particles that do not extend as far upward from the casting plate experience less shear force from the doctor blade at the surface of the tape so that they remain relatively stationary to the surrounding steel powder particles adjacent to the casting surface. Nevertheless, a simple solution is applying an additional sanding step (200 grit) to all PF40, PF60, and PF90 tapes to remove the steel layer until the pore formers are visible.

**Figure 5:**
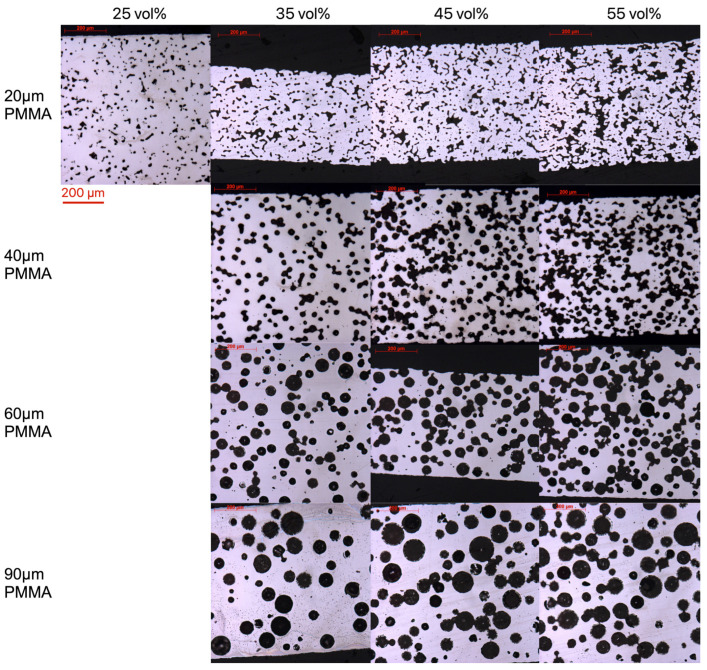
Cross-sectional optical micrographs show a microstructure comparison of PF20 to PF90 substrates.

Cross-sectional images of all substrates are shown in Figure 5. When using PF20 as the pore former, the porosity is isolated, with some granularity at 25% pore former content; as the volume fraction increases, the pore becomes irregular and starts to form a more connected porosity network. The small porosity and steel fingers observed in conventional metal supports start to be more observable as the performer content increases. When using PF40, PF60, and PF90 as the pore former, the individual spherical pores are visible at 35%, and as the pore-former size increases, the pores are more isolated, since the number of pore former decreases with increased particle size. At 55% pore-former content, all samples start to show small steel fingers, formed at the necking between two spherical pores. As the pore-former size increases, the number of steel fingers decreases, and the size increases. Small porosity is only present at the necking between major pores.

### 3.2. Substrate Internal Structure Properties

**Figure 6:**
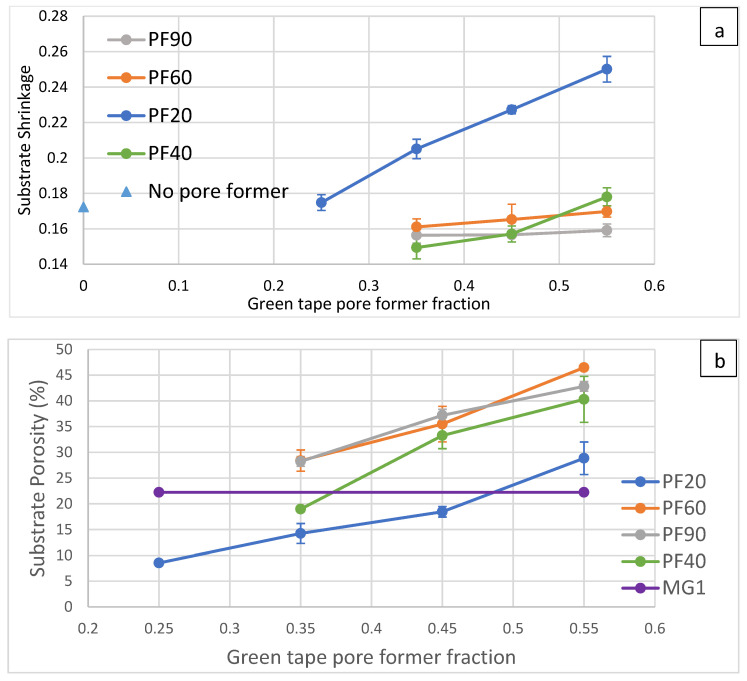

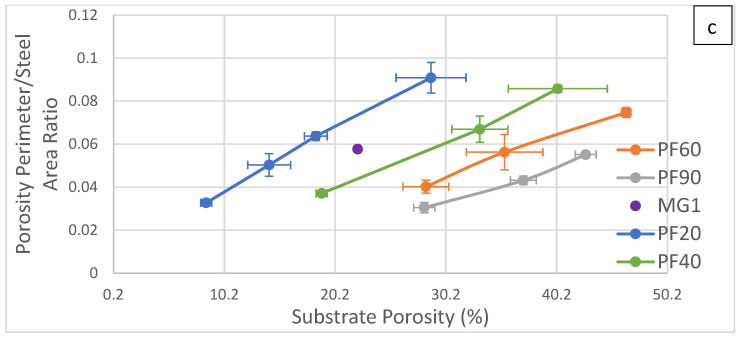
Substrate shrinkage (**a**) and final porosity (**b**) with various pore former sizes and volume fractions. The substrate-porosity-perimeter-to-steel-area ratio and corresponding porosity for all pore-former fractions are shown in (**c**).

Figure 6a shows the shrinkage of various green tape compositions after sintering at 1200 °C for 4 h. Measuring the shrinkage can be helpful in calculating green body dimensions to achieve the desired sintered part dimension. For PF20, the shrinkage increases linearly as the pore-former fraction increases. The lowest shrinkage is slightly larger than the tape without pore former. For larger pore-former sizes like PF60 and PF90, the shrinkages are relatively consistent regardless of the pore former fraction, and they are all smaller than those without pore former. The PF40 results are similar to those of PF60 but have a more significant shrinkage increment with the pore-former volume fraction. The data on higher pore-former contents were not included because the tape becomes very fragile after the binder has been removed.

The different shrinkages between PF20 and larger pore formers imply that the PF20 particles are not large enough to maintain their pore shapes individually, and the PF20 pores in Figure 5 are less spherical than those of PF40 and beyond. This result agrees with the previous study [46,47]: porosity tends to close unless the number of surrounding particles exceeds a critical value. The pore-former size controls the number of particles surrounding the porosity (coordination number). Based on the observed results, the critical number is between the coordination number for the PF20 and PF40 pore-former particles.

The solid-state sintering according to Coble [48] can be divided into three stages: the initial stage of interface and neck formation between solid particles; the intermediate stage of grain growth and grain boundary formation, while pores are still connected as a network; and the final stage of densification when pores are isolated and reduced. To reduce porosity in green bodies, the stress from surface tension (*σ_s_*) which reduces pores should be greater than the stress from interface energy (*σ_i_*) that expands pores. These two stresses can be described as follows [49]:

*σ_s_* = *d*(4*πr*^2^*γ_s_*)/*d*(*r*)/(*4πr*^2^) = 2 *γ_s_*/*r* (7)

*σ_i_* = *d*(*nl*2*γ_i_r*)/*d*(*r*)/(4*πr*^2^) = *n_s_lγ_i_*/(2*πr*^2^) (8)

where *r* is the pore diameter, *γ_s_* is the solid–gas surface tension, *γ_i_* is the solid interface tension, *n_s_* is the coordination number of grains surrounding the pore, and *l* is the arc length of the grain boundary in the solid phase.

When introducing pore formers into a green body, there are two types of pore porosity: pore-former porosity directly coming from pore formers and smaller packing porosity between packing particles. The pore-former porosity radius *r_p_* is directly dependent on the pore-former size, and the packing porosity radius *r_s_* can be approximated as 0.4 of the packing particle radius [50]. As the pore-former size increases while the surrounding particles maintain the same size, the surface tension *σ_s_* decreases at an inverse relation with *r_p_*. The coordination number increases as the pore surface area increases proportionally with *r_p_*^2^. So, the interface stress is relatively consistent because the coordination number in the numerator and *r_p_*^2^ in the dominator increase at the same time.

By proposing using smaller steel particles in this work, when compared to the larger steel particle, *r_s_* will be smaller. So, in the final densifying stage, the amount of material and distance of solid-state diffusion is lesser compared to larger packing particles, and it takes less time to reach the final density.

The substrate porosity and perimeter-to-steel-area ratio are shown in Figure 6b and Figure 6c, respectively, with additional MG1 commercial stainless steel filters as a comparison. This type of support shows a structure similar to the common existing solution, and it is easier to compare them by directly using this support and going through the same measurements with the new supports from this study. The porosity-perimeter-to-steel-area ratio can quantitatively tell how the porosity is distributed. Larger values mean smaller porosity and more steel is exposed to gas, which will make the support more susceptible to dropped permeability after chromium oxide formation and breakaway oxidation after a longer operation time. Overall, PF20 tapes have lower porosity at the same pore-former content, which agrees with the result of the highest shrinkage. PF40 shows transitional behavior between PF20 and larger pore formers, indicating that some particles in the PF40 still cannot form stable pores. The PF60 and PF90 results show similar porosity; these sizes do not have a significant impact on shrinkage or porosity. The porosity-perimeter-to-steel-area ratio in the 2D cross-sectional image approximates the surface-to-volume ratio in the 3D solid, assuming that the porosity is isotropic, based on isotropic shrinkage. From this result, PF60 with 35–45% volume fraction and PF90 with 35–55% volume fraction have equal or lower surface area per volume and more porosity than MG1.

All of the lines shown in Figure 6 are fitted with a linear model to find the slope and R^2^ values; the results are summarized in Table 2. When comparing the slope of shrinkage with different pore formers, there is a decreasing trend, with PF20 having the highest slope of 0.2478, and PF90 having the lowest slope of 0.0138. Since all of the pore formers used have a range of size distribution, the larger the average size, the lower the amount of stable pores created by small pore formers; hence, the shrinkage is less dependent on the pore-former fraction. In the plot of PF90, the second data point is smaller than other data points and the slope is close to 0; this could explain why the R^2^ of PF90 is not very close to 1 like the other three pore formers. The dependency of porosity on pore-former content in green bodies with various pore-former sizes does not show a clear trend with increasing pore-former size. There is a jump from 65 to 106 when the PF20 changes to PF40, and the slope slowly decreases to 73 as the pore-former size increases. The gentle slope of PF20 can be explained by the smaller size; some small porosity is not stable. However, the decreasing trend from PF40 to PF90 is not straightforward. One possible explanation is that for the same volume and mass of pore formers, a smaller pore former has a larger specific area. In a green body, the effective stable pore volume created by pore formers is greater than the pore former’s particle volume. The packing porosity around the pore former should also be considered. This extra volume can be approximated to be proportional to the surface area of individual particles. Since PF40, PF60, and PF90 can all create stable porosity from individual particles, the total volume of stable porosity in PF40 should be greater than PF60, followed by PF90 after accounting for the neighbor packing porosity. This idea can also explain why the shrinkage from those larger pore formers is smaller than that without pore former. The perimeter ratio slope on substrate porosity decrease with increasing pore former sizes can be expected. As shown in Figure 5, the porosity becomes less complicated with increasing pore former sizes.

### 3.3. Substrate Surface Profiles

**Figure 7:**
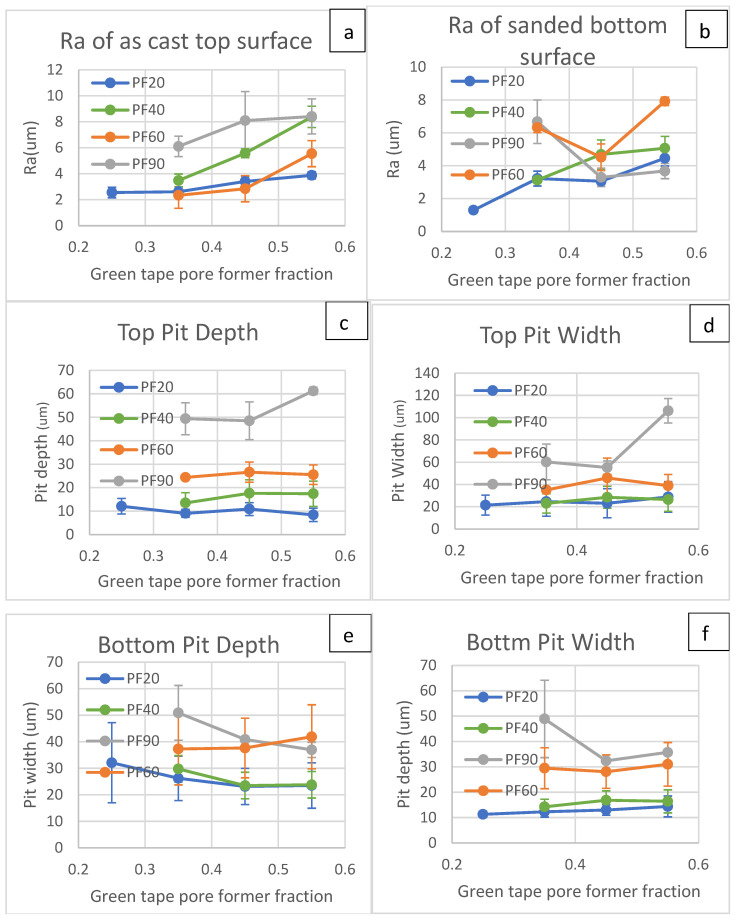

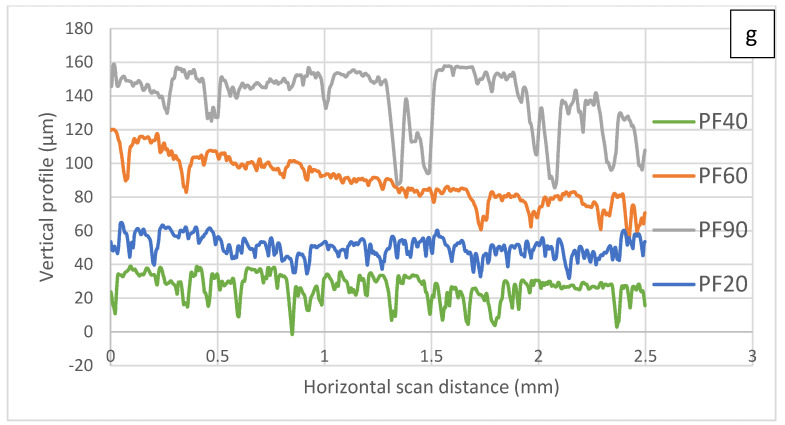
Surface profile analysis results of top-face roughness (**a**), bottom-face roughness (**b**), top-face average pit width (**c**), bottom-face average pit width (**d**), top-face average pit depth (**e**) and bottom-face average pit depth (**f**). The sample top surface profile of four different pore formers at 45 vol% is shown in (**g**).

Figure 7 shows the full results of measured Ra, pit width, and pit depth. Ra is the parameter that determines early plasma deposition efficiency; small Ras yield low deposition efficiency as the incident material gets carried away by plasma gas [51]. A large Ra indicates large surface height variations; in the case of tape cast support, it is often caused by large open pits. Material from plasma spray can get deposited into the pits, causing discontinuity and offsets in the deposited layers. This is crucial for electrolyte, which needs to be gas-tight. Previous work showed that a range between 2 µm and 8 µm can give the desired deposition quality [19].

For the top surface, the Ra of PF60 is about the same as or larger than that of PF20 but smaller than PF40, while PF90 has the highest Ra. The Ra values of the bottom surface do not show a clear relationship to the pore-former fractions for those sanded samples. The greater Ra of PF40 than PF60 can be explained by the larger number of PF40 particles for the same volume fraction, making the Ra higher. The average pit depth and width of the top surface increase as the pore former size increases. However, for the bottom surface, such a relation is not clear, especially for the surface roughness and pit depth, possibly because by sanding the bottom side, it is hard to control tapes to have the material evenly removed. When deciding whether there was sufficient sanding, visual inspection was used to see if there were sufficient PMMA beads exposed. Also, sandpaper is filled with removed powder that can make local roughness different compared to areas sanded by fresh sandpaper. The P220 sandpaper has a nominal size of 53 µm, which explains the PF90’s 35% higher values in Figure 7e,f. Nevertheless, pit width and depth are still in the acceptable range when up to 45 vol% pore former is used.

When comparing the profiles from the top and bottom surfaces, except the PF20 (the unsanded sample), the Ra, pit depth, and width are different from the top surface. For the top pit depth, the measured depth is relatively consistent regardless of the increasing pore-former fraction; the pit width values showed a similar trend, except for PF90 at the 0.55 fraction. The profile from the bottom surface shows much more significant deviations and less pit depth and width consistency across different pore-former fractions for each pore-former size. The differences between the top and bottom surfaces suggest the sanding process modified the surface properties. During the sanding process, we noticed that the sandpaper became less abrasive after the steel powder filled up the space between the sand grits. This greatly affected the material removal rate as well as the scratch depth left on the green tape, which will affect the surface properties. The surface profiles change from site to site as the sandpaper condition changes. For reliable sprayed layers on those substrates, the top surface should be used for deposition. Figure 7g shows the example surface profiles from various pore former sizes; with a larger pore former, there were fewer but wider and deeper pits detected. The large pit depth from the PF20 bottom side at the 0.25 fraction is possible due to a detected pinhole.

### 3.4. Substrate Oxidation and Gas Permeability

**Figure 8:**
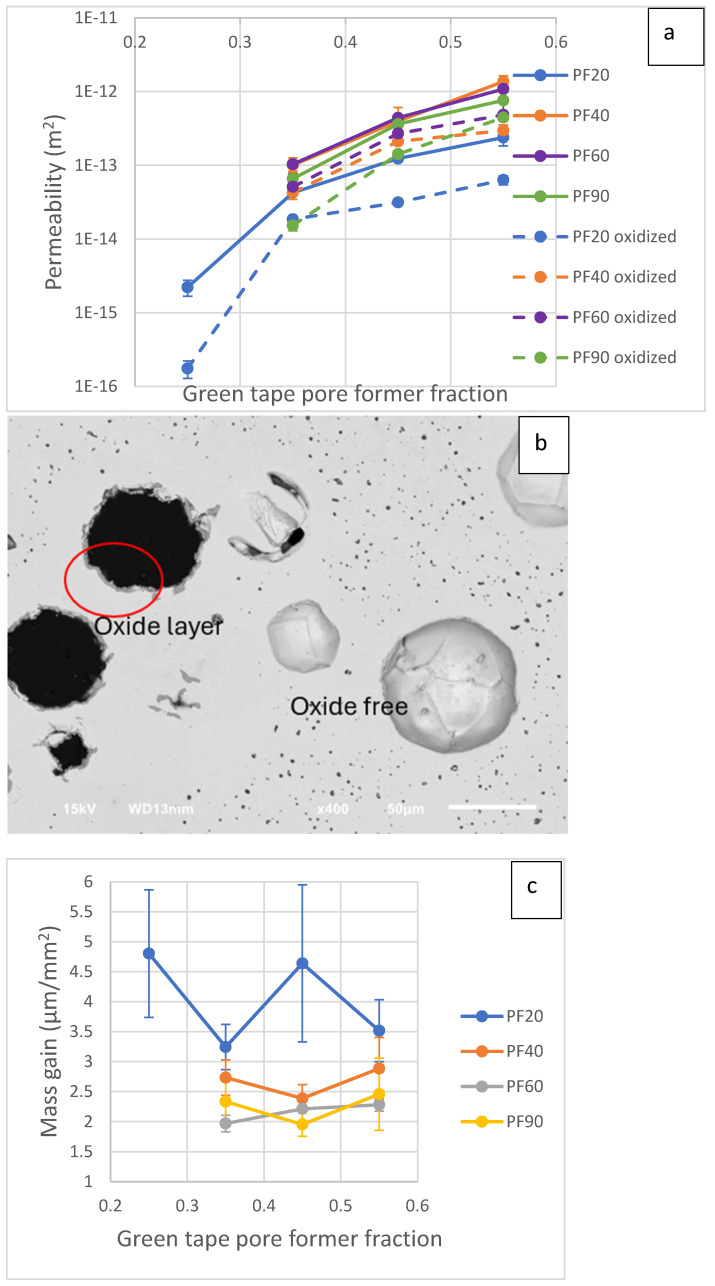
(**a**) Substrate gas permeability before and after oxidation test. (**b**) Backscattered electron image of oxidized 35% PF90 substrate, showing oxide-free closed porosity. (**c**) Normalized mass gain per area with respect to pore former size and fractions.

Figure 8a shows the gas permeability of substrates before and after oxidation. For the raw substrates, PF60 and PF40 are the most permeable substrates; PF90 permeability is slightly lower than that of these two pore formers. Because PF90 has larger particles, the particle count of PF90 is smaller than that of small pore formers, so there is less chance for the pore former to form a connected pore network with PF90, resulting in less permeability. PF20 has less permeability at all pore former contents because the porosity is lower.

Youssef et al. [52] presented a way to calculate the fluid permeability of a porous media. They made the approach by dividing and simplifying the pore network into spherical pores connected with straight channels. In their model, the number and size of the channels mainly control the material permeability. The conductance of fluid through a cylindrical channel is proportional to the tube radius at a power of 4 [52]. The oxide formation with the porosity can effectively reduce the channel diameters available for fluid transport, hence lowering the permeability. For narrow tubes in PF20 and conventional substrates, a decrease of 2 µm from oxide growth can be a large reduction by portion compared to large diameter tubes. The dependency of the radius’ power of 4 further magnifies the impact. So, we can observe there is a larger permeability drop in PF20 compared to larger pore formers in Figure 8a. PF60 has the smallest permeability drop after oxidation; besides the larger pore sizes, a more connected pore network than PF90 can also contribute to higher gas permeability.

By using image analysis to measure the oxide layer thickness, all substrates, including the substrate without pore former, have a similar oxide thickness of about 2 µm, like that shown in Figure 8b. The values are comparable to the reported data of 2 µm at 800 °C for 300 h [53]. This result suggests that all pore-former sizes should give similar results. Also, for the same pore former size with different volume fractions, the normalized mass gains are similar, which validates the image analysis results. Such disagreement between the oxide layer thickness and normalized mass gain requires further study in the future to give a better explanation.

In Figure 8c, the PF20 has the highest unit area mass gain, followed by PF40. PF60 and PF90 give similar values. The actual mass gain for PF90 at 35% should be larger, considering that some closed pores are not oxidized, but the surface-to-volume ratio is based on total porosity. The PF20 mass gain has some large 25% and 45% variations. For those samples, spots of extra oxide formation were observed, raising the average mass gain and deviation. A possible explanation is that some spots have prominent points where breakaway oxidation happened. Some data points from 25% and 45% are close to those of 35% and 55%, with more minor variations.

In conclusion, by evaluating the effect of various spherical PMMA pore-former sizes and volume fractions on gas permeability, surface roughness, pit width and depth, as well as steel surface-to-volume ratio, the PF60 at the 0.45 volume fraction gives the most balanced performance as a metal support for plasma-sprayed solid oxide cells. With this composition, we can achieve an improvement of 1.6 times for porosity and 3 times for gas permeability compared to conventional metal supports. This study would also benefit those who prefer to co-sinter metal supports with electrolytes to retain adequate porosity. Besides the application in solid oxide cells, this study also has the potential to benefit applications such as oxygen separation membranes, catalyst support and metal water filters for improved gas permeability and a reduced surface-to-volume ratio.

## Figures and Tables

**Table 1:** Summarized particle size distributors of powder used for this study.

	d_0.1_ (µm)	d_0.5_ (µm)	d_0.9_ (µm)	Span	Specific Area (m^2^/g)
430L	2.93	5.67	10.72	1.38	0.183
PF20	14.14	19.19	26.21	0.63	0.332
PF40	28.26	37.99	50.91	0.60	0.162
PF60	40.96	59.10	85.21	0.75	0.106
PF90	62.26	94.21	141.2	0.84	0.067

**Table 2:** Summarized linear fit of lines in Figure 6.

Pore Former	Shrinkage		Porosity		Perimeter Ratio	
	slope	R^2^	slope	R^2^	slope	R^2^
PF20	0.2478	0.9944	65.095	0.9614	0.0029	0.9974
PF40	0.1427	0.9331	106.56	0.963	0.0023	0.9958
PF60	0.0437	0.9995	90.397	0.9849	0.0019	0.9933
PF90	0.0138	0.7958	72.877	0.983	0.0017	0.9872

## Data Availability

The raw data supporting the conclusions of this article will be made available by the authors on request.

## Supplementary Materials

The following supporting information can be downloaded at: https://www.mdpi.com/article/10.3390/ma17225408/s1.
