# Supplementary material for: Evaluation of Pore-Former Size and Volume Fraction on Tape Cast Porous 430 Stainless Steel Substrates for Plasma Spraying
Source: Materials (Basel). 2024 Nov 5;17(22):5408. doi: 10.3390/ma17225408 (PMC11595610; doi:10.3390/ma17225408)
Supplement: Supplementary file 1 [file materials-17-05408-s001.zip › materials-3247451-supplementary.pdf]

Supplementary Information

Evaluation of Pore-Former Size and Volume Fraction on Tape Cast Porous 430 Stainless Steel Substrates for Plasma Spraying

Yifei Yan, Olivera Kesler*

Department of Mechanical and Industrial Engineering, University of Toronto, 5 King's College Road, Toronto, ON, M5S 3G8 Canada
* Correspondence: kesler@mie.utoronto.ca

Concentration:	Span :	Uniformity:	Result units:
0.0087 %Vol	1.375	0.466	Volume
**Specific Surface Area:**	**Surface Weighted Mean D[3,2]:**	**Vol. Weighted Mean D[4,3]:**	
0.183 m²/g	4.214 um	6.528 um	

d(0.1): 2.934 um **d(0.5): 5.665 um** **d(0.9): 10.721 um**

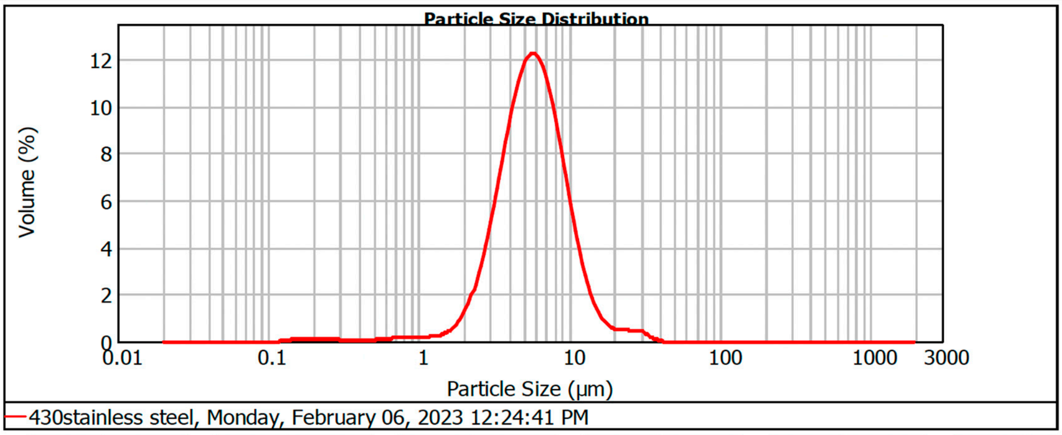

Size (μm)	Volume In %
0.010	0.00
0.011	0.00
0.013	0.00
0.015	0.00
0.017	0.00
0.020	0.00
0.023	0.00
0.026	0.00
0.030	0.00
0.035	0.00
0.040	0.00
0.046	0.00
0.052	0.00
0.060	0.00
0.069	0.00
0.079	0.00
0.091	0.00
0.105	

Size (μm)	Volume In %
0.105	0.00
0.120	0.03
0.138	0.07
0.158	0.10
0.182	0.12
0.209	0.11
0.240	0.09
0.275	0.06
0.316	0.04
0.363	0.03
0.417	0.04
0.479	0.06
0.550	0.09
0.631	0.13
0.724	0.16
0.832	0.17
0.955	0.18
1.096	

Size (μm)	Volume In %
1.096	0.18
1.259	0.22
1.445	0.34
1.660	0.63
1.905	1.17
2.188	2.07
2.512	3.38
2.884	5.04
3.311	6.90
3.802	8.71
4.365	10.17
5.012	10.97
5.754	10.94
6.607	10.08
7.586	8.53
8.710	6.62
10.000	4.65
11.482	

Size (μm)	Volume In %
11.482	2.98
13.183	1.73
15.136	0.97
17.378	0.58
19.953	0.45
22.909	0.43
26.303	0.42
30.200	0.26
34.674	0.09
39.811	0.00
45.709	0.00
52.481	0.00
60.256	0.00
69.183	0.00
79.433	0.00
91.201	0.00
104.713	0.00
120.226	

Size (μm)	Volume In %
120.226	0.00
138.038	0.00
158.489	0.00
181.970	0.00
208.930	0.00
239.883	0.00
275.423	0.00
316.228	0.00
363.078	0.00
416.869	0.00
478.630	0.00
549.541	0.00
630.957	0.00
724.436	0.00
831.764	0.00
954.993	0.00
1096.478	0.00
1258.925	

Size (μm)	Volume In %
1258.925	0.00
1445.440	0.00
1659.587	0.00
1905.461	0.00
2187.762	0.00
2511.886	0.00
2884.032	0.00
3311.311	0.00
3801.894	0.00
4365.158	0.00
5011.872	0.00
5754.399	0.00
6606.934	0.00
7585.776	0.00
8709.636	0.00
10000.000	

Figure S1. Particle size analysis report for 430L powder.

Concentration: 0.0463 %Vol

Span : 0.629

Uniformity: 0.195

Result units: Volume

Specific Surface Area: 0.322 m²/g

Surface Weighted Mean D[3,2]: 18.660 um

Vol. Weighted Mean D[4,3]: 19.738 um

d(0.1): 14.144 um **d(0.5): 19.188 um** **d(0.9): 26.211 um**

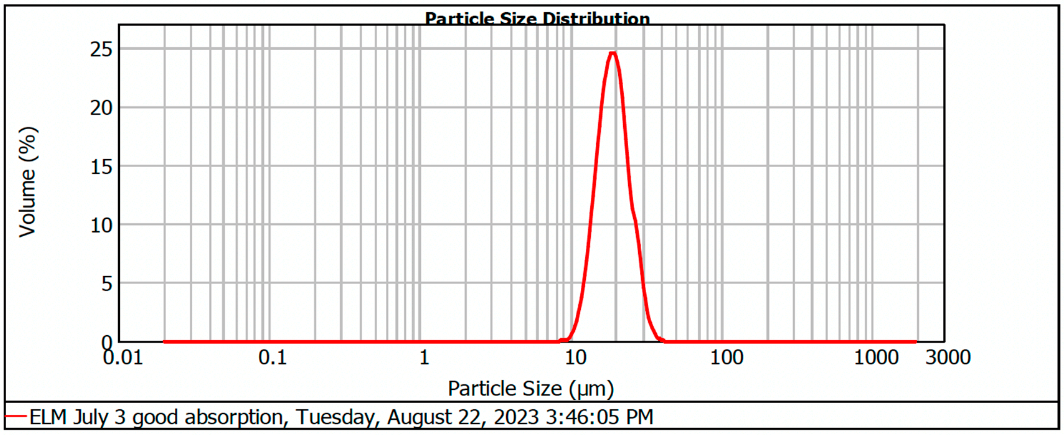

Size (μm)	Volume In %
0.010	0.00
0.011	0.00
0.013	0.00
0.015	0.00
0.017	0.00
0.020	0.00
0.023	0.00
0.026	0.00
0.030	0.00
0.035	0.00
0.040	0.00
0.046	0.00
0.052	0.00
0.060	0.00
0.069	0.00
0.079	0.00
0.091	0.00
0.105	

Size (μm)	Volume In %
0.105	0.00
0.120	0.00
0.138	0.00
0.158	0.00
0.182	0.00
0.209	0.00
0.240	0.00
0.275	0.00
0.316	0.00
0.363	0.00
0.417	0.00
0.479	0.00
0.550	0.00
0.631	0.00
0.724	0.00
0.832	0.00
0.955	0.00
1.096	

Size (μm)	Volume In %
1.096	0.00
1.259	0.00
1.445	0.00
1.660	0.00
1.905	0.00
2.188	0.00
2.512	0.00
2.884	0.00
3.311	0.00
3.802	0.00
4.365	0.00
5.012	0.00
5.754	0.00
6.607	0.00
7.586	0.00
8.710	0.07
10.000	1.04
11.482	

Size (μm)	Volume In %
11.482	4.35
13.183	10.73
15.136	18.06
17.378	22.09
19.953	20.77
22.909	13.14
26.303	7.36
30.200	2.04
34.674	0.35
39.811	0.00
45.709	0.00
52.481	0.00
60.256	0.00
69.183	0.00
79.433	0.00
91.201	0.00
104.713	0.00
120.226	

Size (μm)	Volume In %
120.226	0.00
138.038	0.00
158.489	0.00
181.970	0.00
208.930	0.00
239.883	0.00
275.423	0.00
316.228	0.00
363.078	0.00
416.869	0.00
478.630	0.00
549.541	0.00
630.957	0.00
724.436	0.00
831.764	0.00
954.993	0.00
1096.478	0.00
1258.925	

Size (μm)	Volume In %
1258.925	0.00
1445.440	0.00
1659.587	0.00
1905.461	0.00
2187.762	0.00
2511.886	0.00
2884.032	0.00
3311.311	0.00
3801.894	0.00
4365.158	0.00
5011.872	0.00
5754.399	0.00
6606.934	0.00
7585.776	0.00
8709.636	0.00
10000.000	

Figure S2. Particle size analysis report for PF20 powder.

**Concentration:**<br>0.0885 %Vol	**Span :**<br>0.596	**Uniformity:**<br>0.19	**Result units:**<br>Volume
**Specific Surface Area:**<br>0.162 m²/g	**Surface Weighted Mean D[3,2]:**<br>37.057 um	**Vol. Weighted Mean D[4,3]:**<br>39.037 um	

d(0.1): 28.259 um **d(0.5): 37.989 um** **d(0.9): 50.910 um**

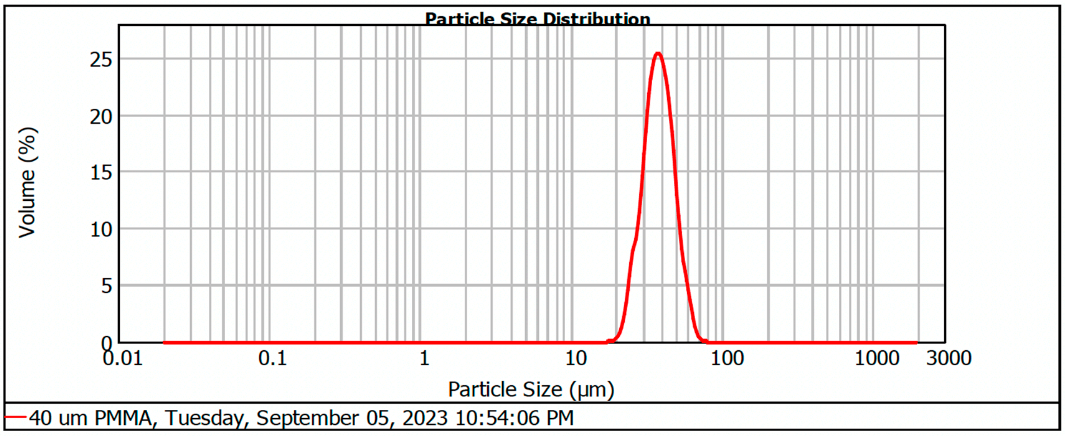

Size (μm)	Volume In %	Size (μm)	Volume In %	Size (μm)	Volume In %	Size (μm)	Volume In %	Size (μm)	Volume In %	Size (μm)	Volume In %
0.010	0.00	0.105	0.00	1.096	0.00	11.482	0.00	120.226	0.00	1258.925	0.00
0.011	0.00	0.120	0.00	1.259	0.00	13.183	0.00	138.038	0.00	1445.440	0.00
0.013	0.00	0.138	0.00	1.445	0.00	15.136	0.00	158.489	0.00	1659.587	0.00
0.015	0.00	0.158	0.00	1.660	0.00	17.378	0.05	181.970	0.00	1905.461	0.00
0.017	0.00	0.182	0.00	1.905	0.00	19.953	0.67	208.930	0.00	2187.762	0.00
0.020	0.00	0.209	0.00	2.188	0.00	22.909	4.73	239.883	0.00	2511.886	0.00
0.023	0.00	0.240	0.00	2.512	0.00	26.303	10.20	275.423	0.00	2884.032	0.00
0.026	0.00	0.275	0.00	2.884	0.00	30.200	19.22	316.228	0.00	3311.311	0.00
0.030	0.00	0.316	0.00	3.311	0.00	34.674	22.92	363.078	0.00	3801.894	0.00
0.035	0.00	0.363	0.00	3.802	0.00	39.811	20.76	416.869	0.00	4365.158	0.00
0.040	0.00	0.417	0.00	4.365	0.00	45.709	13.53	478.630	0.00	5011.872	0.00
0.046	0.00	0.479	0.00	5.012	0.00	52.481	5.95	549.541	0.00	5754.399	0.00
0.052	0.00	0.550	0.00	5.754	0.00	60.256	1.86	630.957	0.00	6606.934	0.00
0.060	0.00	0.631	0.00	6.607	0.00	69.183	0.11	724.436	0.00	7585.776	0.00
0.069	0.00	0.724	0.00	7.586	0.00	79.433	0.00	831.764	0.00	8709.636	0.00
0.079	0.00	0.832	0.00	8.710	0.00	91.201	0.00	954.993	0.00	10000.000	
0.091	0.00	0.955	0.00	10.000	0.00	104.713	0.00	1096.478	0.00		
0.105		1.096		11.482		120.226		1258.925			

Figure S3. Particle size analysis report for PF40 powder.

Concentration:
0.1286 %Vol

Span :
0.749

Uniformity:
0.235

Result units:
Volume

Specific Surface Area:
0.106 m²/g

Surface Weighted Mean D[3,2]:
56.776 um

Vol. Weighted Mean D[4,3]:
61.466 um

d(0.1): 40.955 um **d(0.5):** 59.101 um **d(0.9):** 85.209 um

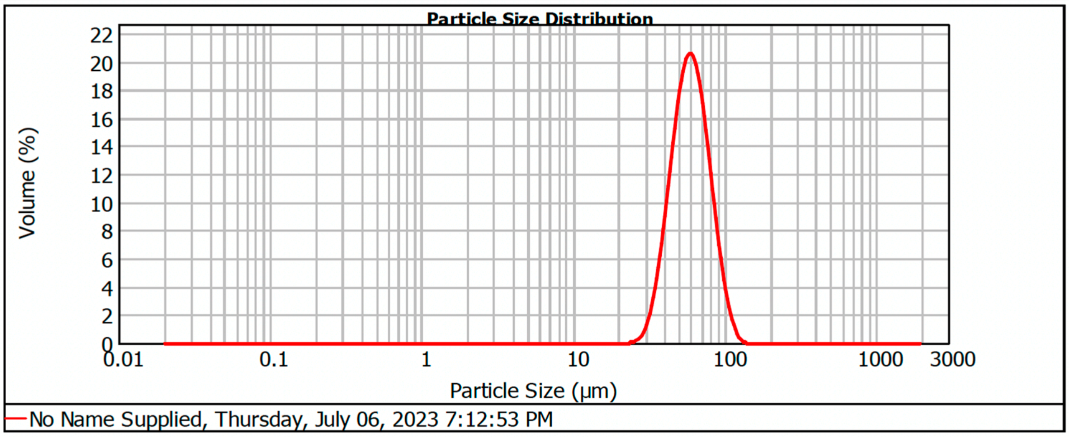

Size (μm)	Volume In %	Size (μm)	Volume In %	Size (μm)	Volume In %	Size (μm)	Volume In %	Size (μm)	Volume In %	Size (μm)	Volume In %
0.010	0.00	0.105	0.00	1.096	0.00	11.482	0.00	120.226	0.18	1258.925	0.00
0.011	0.00	0.120	0.00	1.259	0.00	13.183	0.00	138.038	0.00	1445.440	0.00
0.013	0.00	0.138	0.00	1.445	0.00	15.136	0.00	158.489	0.00	1659.587	0.00
0.015	0.00	0.158	0.00	1.660	0.00	17.378	0.00	181.970	0.00	1905.461	0.00
0.017	0.00	0.182	0.00	1.905	0.00	19.953	0.00	208.930	0.00	2187.762	0.00
0.020	0.00	0.209	0.00	2.188	0.00	22.909	0.02	239.883	0.00	2511.886	0.00
0.023	0.00	0.240	0.00	2.512	0.00	26.303	0.49	275.423	0.00	2884.032	0.00
0.026	0.00	0.275	0.00	2.884	0.00	30.200	2.17	316.228	0.00	3311.311	0.00
0.030	0.00	0.316	0.00	3.311	0.00	34.674	5.60	363.078	0.00	3801.894	0.00
0.035	0.00	0.363	0.00	3.802	0.00	39.811	10.51	416.869	0.00	4365.158	0.00
0.040	0.00	0.417	0.00	4.365	0.00	45.709	15.46	478.630	0.00	5011.872	0.00
0.046	0.00	0.479	0.00	5.012	0.00	52.481	18.39	549.541	0.00	5754.399	0.00
0.052	0.00	0.550	0.00	5.754	0.00	60.256	17.89	630.957	0.00	6606.934	0.00
0.060	0.00	0.631	0.00	6.607	0.00	69.183	14.21	724.436	0.00	7585.776	0.00
0.069	0.00	0.724	0.00	7.586	0.00	79.433	9.05	831.764	0.00	8709.636	0.00
0.079	0.00	0.832	0.00	8.710	0.00	91.201	4.50	954.993	0.00	10000.000	
0.091	0.00	0.955	0.00	10.000	0.00	104.713	1.53	1096.478	0.00		
0.105		1.096		11.482		120.226		1258.925			

Figure S4. Particle size analysis report for PF60 powder.

**Concentration:**	**Span :**	**Uniformity:**	**Result units:**
0.2427 %Vol	0.838	0.262	Volume
**Specific Surface Area:**	**Surface Weighted Mean D[3,2]:**	**Vol. Weighted Mean D[4,3]:**	
0.0671 m²/g	89.430 um	98.570 um	

d(0.1): **62.262** **um** **d(0.5):** **94.206** **um** **d(0.9):** **141.221** **um**

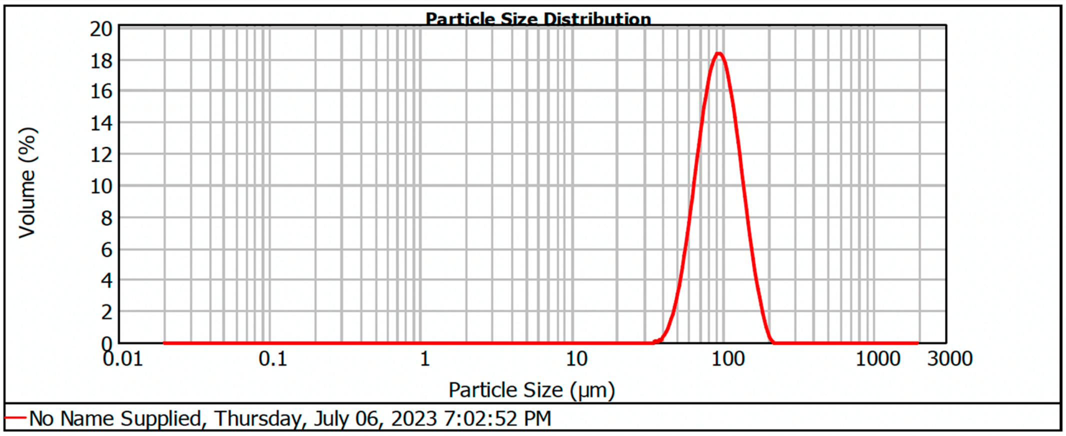

Size (μm)	Volume In %
0.010	0.00
0.011	0.00
0.013	0.00
0.015	0.00
0.017	0.00
0.020	0.00
0.023	0.00
0.026	0.00
0.030	0.00
0.035	0.00
0.040	0.00
0.046	0.00
0.052	0.00
0.060	0.00
0.069	0.00
0.079	0.00
0.091	0.00
0.105	

Size (μm)	Volume In %
0.105	0.00
0.120	0.00
0.138	0.00
0.158	0.00
0.182	0.00
0.209	0.00
0.240	0.00
0.275	0.00
0.316	0.00
0.363	0.00
0.417	0.00
0.479	0.00
0.550	0.00
0.631	0.00
0.724	0.00
0.832	0.00
0.955	0.00
1.096	

Size (μm)	Volume In %
1.096	0.00
1.259	0.00
1.445	0.00
1.660	0.00
1.905	0.00
2.188	0.00
2.512	0.00
2.884	0.00
3.311	0.00
3.802	0.00
4.365	0.00
5.012	0.00
5.754	0.00
6.607	0.00
7.586	0.00
8.710	0.00
10.000	0.00
11.482	

Size (μm)	Volume In %
11.482	0.00
13.183	0.00
15.136	0.00
17.378	0.00
19.953	0.00
22.909	0.00
26.303	0.00
30.200	0.00
34.674	0.05
39.811	0.68
45.709	2.31
52.481	5.19
60.256	9.06
69.183	13.00
79.433	15.83
91.201	16.50
104.713	14.75
120.226	

Size (μm)	Volume In %
120.226	11.21
138.038	7.00
158.489	3.46
181.970	0.97
208.930	0.00
239.883	0.00
275.423	0.00
316.228	0.00
363.078	0.00
416.869	0.00
478.630	0.00
549.541	0.00
630.957	0.00
724.436	0.00
831.764	0.00
954.993	0.00
1096.478	0.00
1258.925	

Size (μm)	Volume In %
1258.925	0.00
1445.440	0.00
1659.587	0.00
1905.461	0.00
2187.762	0.00
2511.886	0.00
2884.032	0.00
3311.311	0.00
3801.894	0.00
4365.158	0.00
5011.872	0.00
5754.399	0.00
6606.934	0.00
7585.776	0.00
8709.636	0.00
10000.000	

Figure S5. Particle size analysis report for PF90 powder.

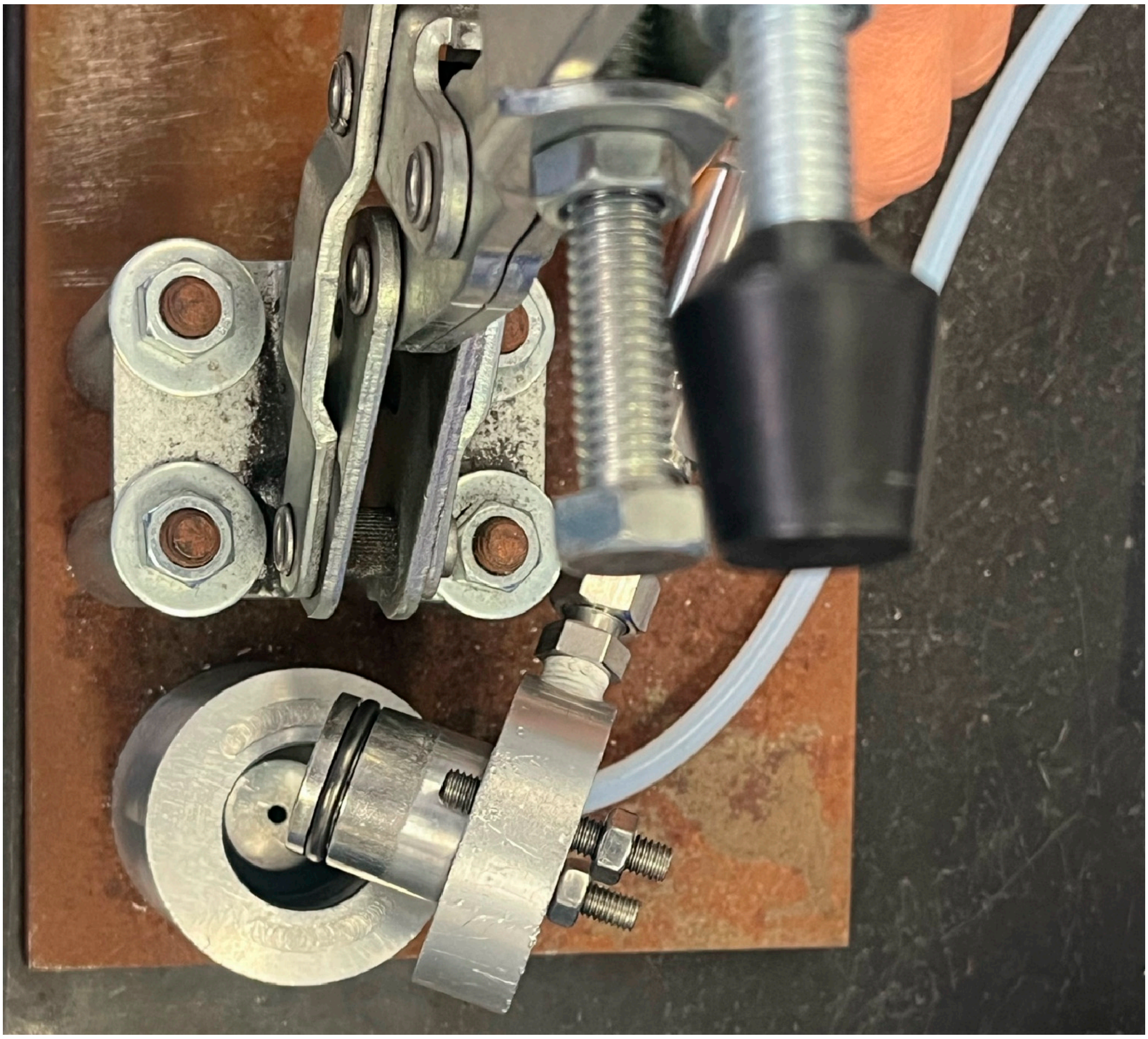

Figure S6. Gas permeability measuring apparatus.

```
    """Python script to analysis surface profile"""
import xml.etree.ElementTree as ET
import os
import statistics

def extract_profile_data(file_path):
    """Extract X and Z coordinates from an XML file."""
    tree = ET.parse(file_path)
    root = tree.getroot()

    x_data = root.findall(".//DataBlock/Data/X")
    z_data = root.findall(".//DataBlock/Data/Z")

    profile = [{"x": float(x_data[i].text), "z": float(z_data[i].text)} for i in range(len(x_data))]
    return profile

def detect_pits(profile):
    """Detect pits based on the Z profile."""
    pits = []
    start_x = None
    start_z = None

    for i, point in enumerate(profile[:-1]):
        current_z = point["z"]
        next_z = profile[i + 1]["z"]

        if current_z > next_z and start_x is None:
            start_x = point["x"]
            start_z = current_z
```

```
        elif current_z < next_z and start_x is not None:
            pits.append({
                "width": point["x"] - start_x,
                "depth": start_z - current_z
            })

            start_x = None
            start_z = None
"""Although the function only tracked the descending part of the profile, the start of a pit is only updated until the descending profile is finished, so the pit width reported here still covers the length of a descending and an ascending profile"""
    return pits


if __name__ == "__main__":
    directory = "empty”
    threshold_depth = 10  # Adjust this value to filter out smaller pits

    pits = []

    for filename in os.listdir(directory):
        if filename.endswith(".xml"):
            profile = extract_profile_data(os.path.join(directory, filename))
            pits += detect_pits(profile)

    major_pits = [pit for pit in pits if pit["depth"] > threshold_depth]
    major_widths = [pit["width"] for pit in major_pits]
    major_depths = [pit["depth"] for pit in major_pits]

    avg_major_width = sum(major_widths) / len(major_widths) if major_widths else 0
```

```
avg_major_depth = sum(major_depths) / len(major_depths) if major_depths else 0
std_dev_width = statistics.stdev(major_widths) if len(major_widths) > 1 else 0
std_dev_depth = statistics.stdev(major_depths) if len(major_depths) > 1 else 0
largest_pit = max(pits, key=lambda pit: pit["depth"]) if pits else {"width": 0, "depth": 0}

print("Average major pit width:", avg_major_width)
print("Average major pit depth:", avg_major_depth)
print("Standard Deviation of major pit width:", std_dev_width)
print("Standard Deviation of major pit depth:", std_dev_depth)
print("Largest pit - Width:", largest_pit["width"], "Depth:", largest_pit["depth"])
```
